# Use of organizational change strategies and personalized agency feedback improves addressing tobacco in behavioral health outpatient treatment settings

**DOI:** 10.3389/frhs.2025.1587795

**Published:** 2025-10-07

**Authors:** Douglas Ziedonis, Robert A. Schnoll, Orrin Myers, Thomas Anthony Chavez, Amy Bachyrycz, Cesar J. Ojeda, Frank T. Leone, Mackenzie Hosie Quinn, Prajakta Adsul

**Affiliations:** ^1^ Department of Psychiatry and Behavioral Sciences, Stanford University, Palo Alto, CA, United States; ^2^ Department of Psychiatry and Behavioral Sciences, University of New Mexico, Albuquerque, NM, United States; ^3^ Department of Psychiatry, School of Medicine, University of California-San Diego, CA, United States; ^4^ Department of Psychiatry, University of Massachusetts Medical School, Worcester, MA, United States; ^5^ Department of Psychiatry, Perelman School of Medicine, University of Pennsylvania, Philadelphia, PA, United States; ^6^ Department of Family and Community Medicine, Health Sciences Center, University of New Mexico, Albuquerque, NM, United States; ^7^ Department of Pharmacy Practice & Administrative Sciences, College of Pharmacy, University of New Mexico, Albuquerque, NM, United States; ^8^ Comprehensive Cancer Center, University of New Mexico, Albuquerque, NM, United States; ^9^ Pulmonary, Allergy, & Critical Care Division, University of Pennsylvania, Philadelphia, PA, United States; ^10^ Division of Epidemiology, Biostatistics, and Preventive Medicine, Department of Internal Medicine, University of New Mexico, Albuquerque, NM, United States; ^11^ Cancer Control and Population Sciences, Comprehensive Cancer Center, University of New Mexico, Albuquerque, NM, United States; ^12^ Center for Advancing Dissemination and Implementation Science, University of New Mexico, Albuquerque, NM, United States

**Keywords:** mental health service, organizational change, implementation strategies, tobacco, addiction, cessation services

## Abstract

**Introduction:**

Implementing evidence-based interventions for tobacco use disorder (TUD) in community mental health agencies is critical, given the low adoption rates of these interventions and the high rates of TUD among patients, contributing to the high morbidity and shortened lifespan of this population. Implementation efforts require enhancing organizational preparedness to integrate these evidence-based interventions.

**Purpose:**

When the Addressing Tobacco Through Organizational Change (ATTOC) model was evaluated in a cluster-randomized trial (with 13 clinics, 610 clients, and 222 staff) and compared with an education-only intervention, ATTOC proved to be better at having more TUD treatment, policies, and staff skills. This paper presents a secondary analysis focusing only on the ATTOC sites, examining whether clinic-level preparedness is associated with increased implementation activities and estimating the combined direct and indirect impact on patient referrals to evidence-based TUD interventions.

**Methods:**

Seven sites applied the ATTOC model over 9 months, with the ATTOC Environmental Scan (ES) conducted at baseline and 3, 6, and 9 months to assess the following: (1) the environment inside and outside the building, (2) staff training and personal tobacco use, (3) clinical TUD services and documentation, and (4) tobacco policies. Summary statistics are provided, and generalized linear mixed model analyses for repeated measures were used to assess time trends and relationships among composite preparedness, activities, and number of referrals.

**Results:**

Over the 9-month period, significant improvements were observed in ES composite preparedness (*p* < 0.001) and individual ES areas (*p* < 0.001 for each). Eight out of 11 ATTOC Dashboard items showed significant changes, including increased number of patients treated (*p* = 0.002); tobacco discussions (*p* = 0.022); provision of educational brochures (*p* = 0.034); referrals to a Nicotine Anonymous group (*p* < 0.001), an in-house wellness or tobacco group (*p* < 0.001), and state quitline (*p* = 0.012); and documentation in treatment plans (*p* = 0.008). Both composite preparedness (*p* = 0.006) and composite activities (*p* < 0.001) were significantly associated with the number of composite referrals.

**Conclusion:**

Significant TUD intervention uptake was found over time through the ATTOC model organizational change intervention and tracking tools.

## Introduction

Tobacco use rates among individuals with serious mental illness (SMI) in the USA are approximately double in clinical settings, with this population accounting for 44% of all cigarettes consumed nationwide (1, 2). This has resulted in significant health disparities in this population, including increased risks of tobacco-associated illnesses such as cancer and respiratory and cardiovascular diseases, along with a 25-year shorter lifespan (2). Attitudes toward smoking have shifted over time, with recent efforts in banning smoking in public and private domains, as smoking contributes to the economic burden exceeding $500 billion annually (3, 4). As a result, this population faces isolating and alienating social consequences, including stigma related to mental health and smoking, which further contributes to poor health outcomes and social disadvantages (3, 5).

Unfortunately, smokers with behavioral health disorders have been historically excluded from smoking cessation clinical trials. However, the EAGLES study (6) has included this population and confirmed that existing evidence-based treatments for tobacco use disorder (TUD) are effective for this population. Despite this evidence, <25% of smokers with SMI receive evidence-based TUD treatment, and mental health clinicians, compared with other specialists, are significantly less likely to address TUD in their practice (7). This may be due to long-held myths among providers, such as the belief that tobacco is used for self-medication, that individuals with SMI are not motivated or able to quit smoking, that psychiatric symptoms will exacerbate if they do quit, and that smoking is a low health priority for this population (8). Transforming the mental healthcare system to integrate and adhere to evidence-based guidelines for TUD treatment is a priority of the National Institute of Mental Health and is a critical component of the national effort to meet Healthy People 2030 target goals for tobacco use (9, 10).

There is growing recognition and emphasis in the field of implementation science to study the mechanics of implementation strategies and measures to advance the field and its impact on health and healthcare (11, 12). Such mechanistic studies are rare in the existing literature and often lack empirical data. This study provides a unique opportunity to delve into the mechanics of a set of implementation strategies and how they interact and act to effect organizational change, in this case, within clinical settings involved in treating TUD among individuals with SMI. For this paper, we use the term tobacco cessation to include smoking cessation and TUD treatment, which includes all tobacco products, e-cigarettes, and other nicotine related products.

### The Addressing Tobacco Through Organizational Change (ATTOC) model

The ATTOC model is an effective approach in promoting organizational change to ensure the provision of TUD treatment in clinical settings (13). This model comprises multiple strategies (Table 1) that support the adoption of evidence-based TUD screening, assessment, and treatment, including psychosocial, pharmacological, and community-based interventions such as the quitline (14). The system changes in ATTOC align with the Agency for Healthcare Research and Quality's (AHRQ) “Systems Change: Treating Tobacco Use and Dependence” based on the Public Health Service (PHS) Clinical Practice Guideline—2008 Update (15). System changes refer to specific strategies that healthcare administrators, managed care organizations, and purchasers of health plans can implement to treat tobacco dependence. These strategies include implementing a tobacco-user identification system; providing training, resources, and feedback; assigning staff to provide tobacco dependence treatment and assessing treatment delivery through performance evaluations; and promoting hospital policies that support and provide tobacco dependence services.

**Table 1:** ATTOC model—implementation strategies.

1. Meetings, calls, and videoconferences to prepare for and implement the intervention 2. On-site consultation and technical assistance, including a baseline and repeated environmental scan (i.e., determination of current patient assessment and treatment; staff smoking, training, and attitudes and beliefs; and evaluation of indoor and outdoor agency spaces for evidence of tobacco use and tobacco-related policies) 3. Formation of the agency's tobacco champion/leadership to support culture and practice change, including the use of a “dashboard” assessment to provide staff with performance feedback 4. Implementation of the agency's change plan to achieve staff and agency goals (e.g., initiation of TUD treatment training, methods of tobacco use assessment, and documentation of treatment plans 5. Formal training in treating tobacco use with monitoring, feedback, and coaching by champions 6. Sustained consultations, including the use of the dashboard assessment to monitor organizational change and provide feedback 7. Web-based supports

The ATTOC model provides a structured approach that links organizational change to improved implementation and outcomes in tobacco cessation by ensuring comprehensive support for quitting tobacco. It facilitates this link through an initial comprehensive assessment of current tobacco use, policies and procedures, tobacco cessation practices and strategies currently being utilized, and areas needing improvement. This model also allows for strategic planning to address identifiable gaps, define specific goals and timelines, and assign responsibilities for implementing tobacco cessation initiatives. Because ATTOC is an evidence-based and validated approach, it can help secure organizational leadership to gain commitment for implementation and sustainability. Leaders are influential for championing the areas discussed in the initial assessment, promoting tobacco-free policies and procedures, allocating resources, and creating a culture and behavioral change that prioritizes tobacco cessation.

Most importantly, the ATTOC model allows for more integrated care, which includes tobacco cessation interventions as routine and standard care, ensuring that all patients are screened for tobacco use and offered cessation support. It also allows for the support of resources that are readily available and facilitates improved patient access to educational materials, counseling services, patient monitoring, pharmacotherapy, and follow-up support.

Possible outcomes that may benefit from the ATTOC model include increased quit rates, a healthier environment, enhanced and more integrated patient care, patient cost savings, and an organizational culture and behavioral shift that makes tobacco cessation both a norm and a priority, with sustained efforts and long-term success in reducing tobacco use. By systematically addressing tobacco use through organizational change, the ATTOC model ensures that tobacco cessation efforts are comprehensive, integrated, and effective, leading to significant improvements in implementation and outcomes.

This approach utilizes core strategies, steps, and phases to help create organizational change. Tobacco cessation interventions are commonly influenced by attitudes and theories of what behavior change should look like, as well as other barriers to tobacco cessation efforts, including staffing limitations, lack of training, and insufficient reimbursement for TUD treatment services (16). Although it has been noted that resistance to change can be observed in both patients and staff, it allows observers to note the systemic nature of these barriers and suggests that organizational change is warranted for success (13, 17). Several studies have documented the impact of the ATTOC intervention and organizational changes on both healthcare teams and patients (13, 18), including building on related approaches and studies (19–21).

Our cluster-randomized trial (18), involving 13 clinics, 610 clients, and 222 staff, compared the ATTOC model to an education-only intervention. Clients at the ATTOC sites reported more TUD treatment from providers at Weeks 12 and 24 (*p*s < 0.05) and more TUD treatment and policies from clinics at Weeks 12, 24, 36, and 52 (*p*s < 0.05). The ATTOC staff reported higher skill levels in TUD treatment at Week 36 (*p* = 0.05). Given the promising findings for the ATTOC intervention in this study, this secondary analysis focuses on the implementation process and intervention tools used across the seven ATTOC sites.

We conceptualize organizational culture as the usual set of expectations and norms that influence behavior within an organization, which includes the historical values and collective experiences of the organization, and the usual ways that things are done (22, 23). The champions within organizations can be described as changing the organizational culture by addressing tobacco use, which counters norms such as not addressing tobacco use, giving cigarettes as a reward for good behavior, allowing smoking on the campus, and believing that tobacco use is an unimportant matter to address.

Implementation science focuses on identifying approaches to integrate evidence-based treatments and interventions into clinical settings in an effective manner. Evidence-based interventions, however, take, on average, approximately 17 years to become integrated into routine practice (24). Implementation science provides an overarching approach that considers the problem to be addressed in which population, what evidence-based treatments and interventions should be integrated, how organizations can be supported to develop the needed support, what organizational change methods might be used to support the leadership and staff, how to ensure sustainability of the change, and how to assess the effectiveness of the change. There is growing recognition in implementation research to allow for a distinct focus on organizational change (25–27). Despite the growing literature around the determinants of organizational change, few models actually provide a deeper understanding of the process of organizational change.

The ATTOC model is a process model specifically focused on healthcare organizations and is guided by the organization development theory (28–31). It provides guiding principles for the implementation of system-wide interventions using behavioral science strategies. This model provides a generalizable, validated set of strategies for use in healthcare settings. Although it is a unique process model, it aligns with the implementation outcome framework, proposed by Proctor et al. (32), in which they set the implementation outcomes in the pathway to achieving service and client health outcomes. We propose that the ATTOC model provides a set of strategies that impact implementation outcomes, especially in the context of organizational change. Indeed, in a recent review, Proctor et al. called for an increased focus on organizational processes and testing mechanistic pathways for implementation outcomes (33). Following this model, we specified our implementation outcomes as the preparedness and the activities undertaken at the organizational level, culminating in the referrals provided to TUD patients.

In this report, we examine the changes at the organizational level following the implementation of the ATTOC model within community mental health agencies to address TUD patients. We hypothesized that implementation indicators collected via the ATTOC Environmental Scan (ES) and the ATTOC Dashboard would increase over time. We next hypothesized that combined environmental, staff, and patient preparedness for the intervention and TUD patient activities are potential mechanistic pathways that would be positively associated with increases in patient referrals. We also investigate whether preparedness is associated with improvement in the number of activities and estimate the combined direct and indirect impact on referrals.

## Materials and methods

Data were collected from clinical staff and leaders who participated in the parent trial (ClinicalTrials.gov ID: NCT02849652). The primary findings from this trial were published elsewhere (18). This secondary analysis examines the ATTOC sites’ organizational changes in depth to better understand the intervention and key effective components. Community mental health clinics (CMHCs) within Philadelphia's Community Behavioral Health system were randomized to one of the two approaches (training alone vs. training and ATTOC). Sites were eligible for this trial if they had an electronic health record, provided access to prescription data (tobacco use treatments), and could enroll at least 12 staff members in the clinical trial. A complete description of the study procedures is reported elsewhere (34).

This report includes data only from seven ATTOC sites. These agencies varied in size, with 28–140 clinicians, 110–1,500 patients, and 60%–80% minorities (patients). The measures of the ATTOC Environmental Scan (baseline and 3, 6, and 9 months) and the ATTOC Dashboard (monthly) were used at these ATTOC intervention sites, which also provided feedback to the clinicians and leadership.

The Institutional Review Boards of the University of Pennsylvania and the City of Philadelphia provided approval for the trial. Following randomization at the level of the CMHC, research personnel attended clinics to enroll staff prior to the intervention. Interested and eligible staff provided informed consent and completed a baseline self-report assessment.

### ATTOC model intervention

The ATTOC model proposes a series of 10 steps, in three distinct phases of (1) preparing and organizing for change; (2) changing, integrating, and adapting; and (3) documenting, monitoring, and sustaining. Within the three phases is a series of 10 steps to guide healthcare delivery sites through organizational change. These phases and steps are flexible to accommodate the unique needs, barriers, and resources of the site implementing change, and organizations are encouraged to consider starting at the point that best matches their context.

During the 9-month ATTOC intervention, seven core strategies (35) were used by the ATTOC intervention consultants with the agencies for implementation, as shown in Table 1. Staff training of evidence-based interventions includes the provision of the AHRQ guideline document, Clinical Guidelines and Recommendations, and an Internet link for training materials on TUD assessment and treatment (14). In addition, the goal of reducing tobacco use on the campus (tobacco-free or restricted use) was an evidence-based public health strategy to support clinical evidence-based practices. The primary ATTOC activities occurred in 10 sessions (2 of which were in-person and on-site visits, and 8 were through videoconference/telephone). Each site set up unique and specific goals in three critical areas: (1) related to their patient care and treatment, (2) staff training and addressing staff smoking, and (3) the agency's campus becoming tobacco-free in total or in a restricted manner. Ongoing consultations were provided through videoconferencing to address any concerns, obstacles, or problems that may emerge during implementation. In addition, site staff were provided with access to an interactive website that provides information, tools, and materials for training and treatment. Site champions were encouraged to use the dashboard assessment of clinician and agency performance to provide formal performance feedback.

In Phase 2 (Weeks 4, 8, and 12), activities supported the implementation of the change plans. These activities included clinical training, TUD treatment service development, and environmental changes.

### Environmental Scan

The Environmental Scan is an assessment tool used at baseline and at 3, 6, and 9 months post-baseline to assess four areas of preparedness of a health organization in addressing TUD: (1) environmental preparedness; (2) patient care preparedness; (3) staff competency, smoking patterns, and attitude; and (4) tobacco policies and procedures (see Table 2). All preparedness measures were scored from 1 to 5: not prepared (1), minimally prepared (2), moderately prepared (3), highly prepared (4), very highly prepared (5). This assessment was performed by the ATTOC intervention consulting team with multiple assessors doing independent scoring (with excellent reliability across assessors through consensus and only a few times an initial deviation greater than one point). Information was gathered through meetings with agency leaders, staff, and patients; review of policies; review of website and social media; walkthroughs of the campus (outside and inside the building); and patient chart reviews.

**Table 2:** ATTOC Environmental Scan components.

I A. Outdoor preparedness score—for tobacco-free campus or restricted use campus				
No restrictions (1)	Minimally (2)	Moderately(3)	Highly (4)	Very highly (5)
Outdoor assessment rating key:				
1. No visible signage present; cigarette butts on the ground outside the building; people seen smoking in close proximity to the building 2. Minimal level of signage present and visible (e.g., only by the main entrance); no messaging distinguishing smoking from non-smoking areas; some cigarette butts strewn on the ground, a few people seen smoking, evidence of smoking by the “no smoking” signs 3. Moderate level of signage present and visible (e.g., front door, back door); minimal messaging distinguishing smoking from non-smoking areas; subtle signs of smoking outside, e.g., only a few cigarette butts, rare observation of persons smoking outside 4. High level of signage present and visible in most of the key areas (entry doors, parking lot) and with messaging distinguishing smoking from non-smoking areas; no evidence of persons smoking in non-designated areas 5. All non-smoking areas designated with visible and clear signage containing comprehensive, agency-specific messaging, e.g., written in large print, bilingual, with the agency’s logo. No designated smoking areas and no evidence of smoking anywhere on the grounds				
I B. Indoor preparedness score				
Not prepared (1)	Minimally (2)	Moderately (3)	Highly (4)	Very highly (5)
Indoor assessment preparedness rating key:				
1. No visible signage present, no client information provided in the waiting area, no information for staff in staff-designated areas. There is a designated smoking area inside the building. 2. Minimal signage and few pertinent informational materials present in high-traffic areas (e.g., by the registration desk, elevator, hallways, waiting rooms, and restrooms) and staff-designated areas (e.g., mailboxes, lunch room, by photocopy machine, and meeting rooms). There is no designated smoking area in the building; however, despite signage, one might detect evidence of smoking. 3. Moderate level of signage present and visible, with a moderate amount of pertinent tobacco and/or wellness information in high-traffic areas (waiting rooms, hallways, meeting rooms, kitchens/cafeterias, etc.). 4. High level of signage present and visible, with adequate tobacco/wellness materials in high-traffic areas (waiting rooms, hallways, meeting rooms, kitchens/cafeterias, etc.). Smoking is not permitted in the building, and there are no signs of smoking taking place. 5. All client and staff areas are designated with visible and clear signage, and tobacco/wellness information, brochures, and posters are freely available and displayed. There are age-appropriate and culturally appropriate materials, and there is absolutely no evidence of smoking anywhere inside the building.				
I C. Agency website and social media preparedness score				
Not prepared (1)	Minimally (2)	Moderately (3)	Highly (4)	Very highly (5)
1. The agency has no tobacco information/resources listed on its website and no presence on social media. 2. The agency has made a commitment to include tobacco information/resources on its website and social media posts. 3. The agency website contains some tobacco information/resources and some presence on social media. 4. The agency website contains a moderate amount of tobacco information/resources and is committed to adding more relevant information. The agency has a moderate presence on social media. 5. The agency website lists agency, community, and state tobacco services and resources. The agency lists its tobacco policy on the website and has a presence on social media promoting tobacco services and resources.				
NA^0^ The agency currently does *not* have a website.				
I D. Billing preparedness score				
Not prepared (1)	Minimally (2)	Moderately (3)	Highly (4)	Very highly (5)
1. The agency does not bill for TUD treatment services or CO tests. 2. The agency occasionally bills for TUD treatment services *or* CO tests, not both. 3. The agency occasionally bills for TUD treatment services *and* CO tests. 4. The agency often bills for TUD treatment services and CO tests. 5. The agency bills for almost all TUD treatment services provided and CO tests performed.				
NA^0^ The agency does *not* provide TUD treatment services or CO testing.				
NA^1^ Under the agency’s current funding structure, they are *not* permitted to bill separately for TUD treatment services provided or CO tests.				
I. Overall environment preparedness score				
Not prepared (1)	Minimally (2)	Moderately (3)	Highly (4)	Very highly (5)
The overall environment preparedness score is an average of the four subscales: outdoor, indoor, website and social media, and billing.				
II A. Patient assessment and treatment planning preparedness score				
Not prepared (1)	Minimally (2)	Moderately (3)	Highly (4)	Very highly (5)
Client assessment and treatment planning preparedness rating key (based on at least five reviewed client/client charts):				
1. There is no indication of tobacco use/nicotine assessment in the chart. 2. Nicotine/tobacco use history is documented alongside other substances of abuse for <50% of reviewed charts (i.e., age of onset, and severity of use). It is not included in the diagnostic formulation or treatment plan. 3. Nicotine/tobacco use history is documented alongside other substances of abuse for >50% of reviewed charts. It is not included in the diagnostic formulation or treatment plan. 4. Nicotine/tobacco use history is documented alongside other substances of abuse for >50% of reviewed charts. It is included in the diagnostic formulation and treatment plan. 5. Charts routinely include information about nicotine/tobacco use history, diagnostic formulation, motivation level, treatment plan, and progress notes. Intervention approaches, e.g., CO meter readings, medication assistance, and behavioral interventions, are noted in the chart.				
II B. Patient medication treatment preparedness score				
Not prepared (1)	Minimally (2)	Moderately (3)	Highly (4)	Very highly (5)
Client medication treatment preparedness rating key (preferably based on at least five reviewed client charts):				
1. No on-site prescribers, and there is no partnering with outside prescribers (e.g., primary care provider (PCP)) or other resources (e.g., quitline). 2. Referrals to outside groups/resources (e.g., Nic A) happen rarely, as does internal prescribing with documentation. 3. Referrals to outside groups/resources happen on occasion, as does internal prescribing with documentation. 4. Well-described partnerships with outside prescribers; refer frequently to outside prescribing partners; frequent on-site prescribing with good documentation of medications. 5. Prescribers are on-site [e.g., medical doctors (MDs) and nurses]; they prescribe the Food and Drug Administration-approved medications very frequently with good documentation of medications.				
II C. Patient psychosocial treatment preparedness score				
Not prepared (1)	Minimally (2)	Moderately (3)	Highly (4)	Very highly (5)
Client psychosocial treatment preparedness rating key (preferably based on at least five reviewed client charts):				
1. No individual or group treatment offered, no encouragement to participate, TUD treatment not integrated into other treatment or care. 2. The general tobacco education group offered with minimal content or expertise regarding cessation. 3. Individual and/or group TUD treatment is offered at the agency, with some indication of specific treatment goals and desired outcomes. 4. Individual and/or group TUD treatment offered at the agency, with well-documented treatment goals and desired outcomes noted in treatment notes and in discharge summary; motivation levels of clients are not assessed, there is no integration with community resources and meetings. 5. Integrated individual and/or group TUD treatment is offered to clients/clients either on-site or in the community and is matched to motivational level.				
II. Overall patient care preparedness score				
Not prepared (1)	Minimally (2)	Moderately (3)	Highly (4)	Very highly (5)
The overall patient care preparedness score is an average of the three subscales: patient assessment and treatment, patient medication treatment, and patient psychosocial treatment.				
III A. Staff are trained and the agency has MDs/registered nurses(RNs) and TUD treatment specialists preparedness score				
Not prepared (1)	Minimally (2)	Moderately (3)	Highly (4)	Very highly (5)
Staff training preparedness rating key:				
1. Tobacco training is not offered, and staff are not trained. 2. No designated staff person assigned to provide TUD treatment for clients. Treatment is offered by some staff with unknown levels of expertise. 3. Designated staff person at agency assigned to provide TUD treatment for clients, but the staff person does not possess any special certification (e.g., tobacco treatment specialist (TTS)). There are no prescribers with training in prescribing tobacco cessation aides. 4. TUD treatment is offered by a certified TUD treatment specialist. There are some prescribers with training in prescribing tobacco cessation aides. 5. All staff have received baseline training on how to work with clients who smoke. TUD treatment is offered by a certified TUD treatment specialist. Prescribers are trained in prescribing tobacco cessation aides. Ongoing training on TUD treatment is offered and supported.				
III B. Staff attitude about addressing tobacco preparedness score				
Not prepared (1)	Minimally (2)	Moderately (3)	Highly (4)	Very highly (5)
Staff attitude about addressing tobacco preparedness rating key:				
1. Resistive staff presenting numerous barriers to addressing tobacco through organizational change. 2. Mixed staff attitude about addressing tobacco through organizational change; some are in support, some in opposition. 3. Staff are supportive of addressing tobacco through organizational change due only to the fact they are being told to do so by their leadership. 4. Staff are engaged and supportive of addressing tobacco through organizational change, though they do not feel qualified or able to do so. 5. Staff are engaged and supportive of addressing tobacco through organizational change. Staff feel competent to contribute to the agency change plan in their work.				
III C. Staff recovery is encouraged and supported preparedness score				
Not prepared (1)	Minimally (2)	Moderately (3)	Highly (4)	Very highly (5)
Staff recovery encouragement preparedness rating key:				
1. No services or encouragement offered for staff tobacco recovery. 2. Staff tobacco recovery is encouraged by agency leadership, but there are no active TUD treatment services to assist staff at the agency. 3. Staff tobacco recovery is covered by the agency’s insurance plan(s), but no on-site services or encouragement are offered. 4. TUD treatment services are offered and communicated to staff. 5. The agency assists in cost-reducing measures for services. Services consist of counseling, tobacco cessation aides, and evidence-based treatment.				
III. Overall staff competency, smoking, and attitude preparedness score				
Not prepared (1)	Minimally (2)	Moderately (3)	Highly (4)	Very highly (5)
The overall staff competency, smoking, and attitude preparedness score is an average of the three subscales: staff are trained, the agency has MDs/RNs and TUD treatment specialists, and staff attitude about addressing tobacco/staff recovery is encouraged and supported.				
IV A. Campus environment policy and procedure preparedness score				
Not prepared (1)	Minimally (2)	Moderately (3)	Highly (4)	Very highly (5)
Policies on tobacco control—environment preparedness rating key:				
1. No existing polices or statement of procedures (SOPs) on tobacco control within the agency. 2. Smoking and non-smoking areas present at the agency. No policy distinguishing these two areas. 3. Smoking and non-smoking areas present at the agency. Clearly distinguished by an agency policy. 4. Clear and enforced tobacco control policy at the agency. The agency has partially tobacco-free grounds. 5. Clear and enforced tobacco control policy at the agency. The agency has completely tobacco-free grounds.				
IV B. Staff policy and procedure preparedness score				
Not prepared (1)	Minimally (2)	Moderately (3)	Highly (4)	Very highly (5)
Policies on tobacco control—staff preparedness rating key:				
1. No existing polices or SOPs on staff tobacco use within the agency. 2. A vague policy is present at the agency. Poor staff compliance with policy. No clear course of action for violations. 3. A somewhat detailed policy present at the agency, supported but not enforced by agency leadership with moderate staff adherence. 4. Direct tobacco control policy at the agency outlining expectations for staff about tobacco use (including disciplinary actions), somewhat enforced by agency leadership with demonstrated staff adherence. No TUD treatment services available. 5. Direct tobacco control policy at the agency outlining expectations for staff about tobacco use (including disciplinary actions) with demonstrated enforcement by agency leadership and strong staff adherence. Staff treatment services are available.				
IV C. Patient policy and procedure preparedness score				
Not prepared (1)	Minimally (2)	Moderately (3)	Highly (4)	Very highly (5)
Policies on tobacco control—client care preparedness rating key:				
1. No existing polices or SOPs integrating tobacco control into client care. 2. A vague policy is present at the agency. No linkage with other protocols or guidelines. Minimal documentation in client records. 3. A somewhat detailed policy present at the agency. Minimal linkage with other protocols or guidelines. Moderate documentation in client records. 4. Detailed policy present at the agency. Some linkage with other protocols or guidelines. Sufficient documentation in client records. 5. Direct tobacco control policy for client care in place. Integration into client care. Detailed documentation in client records.				
IV. Overall tobacco policy and procedure preparedness score				
Not prepared (1)	Minimally (2)	Moderately (3)	Highly (4)	Very highly (5)
The overall tobacco policy and procedure preparedness score is an average of the three subscales: campus environment policy and procedure, staff policy and procedure, and patient policy and procedure.				

Environmental preparedness was evaluated through observations of outdoor and indoor signage, smoking areas, the presence of cigarette butts, and individuals smoking. Signage may include “no smoking” posters and signs that designate smoking and non-smoking areas. An agency that has all non-smoking areas designated with visible and clear signage containing comprehensive, agency-specific messaging (e.g., written in large print, bilingual, and with the agency's logo) and has no evidence of smoking anywhere on the grounds would be considered optimally prepared. Website, social media, use of a carbon monoxide (CO) meter, and billing for TUD services were also considered in this assessment.

Patient care preparedness was evaluated by assessing patient charts, with an average, in this study, of five charts per assessment scan. A well-prepared agency would have documentation of a patient's screening and assessment of tobacco use, a diagnosis of TUD when necessary, motivational level of change, and a treatment plan. An agency would also have either a medication prescriber in place within the agency or in partnership with an external prescriber. Finally, individual or group psychosocial treatments offered on-site and matched to a client's motivational level should be indicated, including referrals to community resources such as quitlines, phone apps, Nicotine Anonymous (Nic A) meetings, or websites.

Staff competency, smoking patterns, and attitude toward tobacco use in general and the staff's own smoking patterns were assessed using a 15-item questionnaire. This scale assesses the staff's own tobacco use, attitude about addressing tobacco in treatment, and prior training. This *staff-level* assessment focuses on readiness to change of the staff and the common barriers to change: staff smoking and use of other tobacco products, level of training on TUD treatment, and their confidence in treating tobacco. Information for this scale was collected from conversations with staff and leadership and by reviewing agency documents and policies.

Finally, tobacco policies and procedures were reviewed to assess policies regarding tobacco use (e.g., restricted use, tobacco-free campus), services provided to patients, and support for staff TUD recovery. This assessment examines if there is dedication to a tobacco-free campus not only in discourse, but in clearly delineated policies and whether these are enforced. A comprehensive TUD policy and standard operating procedures would include descriptions of and how to integrate assessments, use of a CO meter, patient education, diagnosis, treatment planning, treatment access, and detailed documentation of such operations is expected for a well-prepared agency.

The Environmental Scan information and scoring were conducted independently by multiple ATTOC intervention consultants at each assessment time point, and individual findings were reviewed together to compare findings and determine a final version of the ES assessment and the scoring for each of the four areas. The four preparedness areas were scored on a range of 1–5, with a score of 5 marking excellence in preparedness. A table of anchoring points had been developed for this scoring purpose. The four individual scores were summed to provide an overall preparedness score. The findings and scoring were reviewed with the local agency champion and leaders after each ES was completed. In addition, an overall composite preparedness score was calculated by averaging the environmental preparedness score, the patient preparedness score, and the staff competency preparedness score. These three ES scores were significantly correlated (Pearson correlation >0.65 for all) with a standardized Cronbach's *α* = 0.89. We hypothesized that this composite measure can be used to model the association between preparedness and patient referrals during the study.

The Environmental Scan was developed empirically in the process of helping over 100 organizations to adopt evidence-based TUD treatments and restrict tobacco use on their campus. The scan was a comprehensive assessment of the organization that was given to the leadership as feedback on their baseline and then ongoing progress. Each scan had two or three raters who engaged in the same activity, and then the ratings were compared for concordance in evaluation. This process helped refine the instrument for the study. For this paper, we used composite variables and assessed correlations and Cronbach's *α* values to assess the reliability of the proposed composite measures for preparedness, referrals, and activities, described above.

### ATTOC Dashboard

The ATTOC Dashboard is a one-page summary of the agency clinician behaviors related to the implementation of 11 TUD patient activities, documentation, and staff training. This monthly assessment is based on staff self-reports and chart reviews. The dashboard aggregated individual clinician data and summarized information such as patient caseload number, average amount of time spent with each patient and time spent on tobacco discussion, and the number of patients who received an educational brochure, undwerwent a CO test, and/or were referred to a Nic A group, an in-house wellness or tobacco group, state's quitline, or for TUD medications. Documentation of TUD and attendance at staff training were also assessed. Table 3 summarizes this information. A composite variable to represent patient care activities was created by averaging three items: the number of consumers spoken to about tobacco cessation, the number of brochures handed out, and the number of consumers who received a CO test. Variables in the composite activity measure were significantly correlated *p* < 0.03 (Pearson correlations between 0.89 and 0.31) and had a standardized Cronbach's *α* = 0.76. A composite referrals variable was computed by averaging four dashboard items: number of referrals to a Nic A group, number of referrals to an in-house wellness or tobacco group, number of referrals to the state's quitline, and number of consumers assisted in obtaining smoking cessation medications (Table 3). The composite referral variable had a standardized Cronbach's *α* = 0.89 with Pearson correlations among variables ≥0.47 and *p* < 0.001.

**Table 3:** ATTOC Dashboard—patient care related items.

Item	Description	Scoring	Composite variable
1	Number of consumers in your caseload	Count	
2	Number of consumers you’ve spoken to about tobacco cessation	Count	Activities
3	Average amount of time spent with each consumer in hours	Estimated hours	
4	Number of brochures handed out	Count	Activities
5	Number of consumers receiving a CO test	Count	Activities
6	Number of referrals to a Nic A group	Count	Referrals
7	Number of referrals to an in-house wellness or tobacco group	Count	Referrals
8	Number of referrals to the state's quitline	Count	Referrals
9	Number of consumers you helped to get smoking cessation medications	Count	Referrals
10	Staff training events (did you attend)	0 no, 1 yes	
11	Tobacco interventions logged in the treatment or service plan	0 no, 1 some, 2 all	

### Data analysis

All Environmental Scan and ATTOC Dashboard data used in analyses are clinic-level data collected over time. Summary statistics were calculated for the four key Environmental Scan areas, for the composite preparedness score (range from 1, not prepared, to 5, highly prepared; Table 2), for continuous dashboard items, and for the composite activities and referrals scores (Table 3). Frequencies and percentages for categorical dashboard items (staff attendance at training events, tobacco interventions logged) were computed by week.

Generalized linear mixed model analyses for repeated measures were used to assess time trends for Environmental Scan and for ATTOC Dashboard items. The models assumed normally distributed error distributions except for a binary dashboard item for attendance at staff training (logistic model) and a three-level ordinal score for logging tobacco interventions into treatment or service plans (0 none, 1 some, or 2 all; Poisson model). We fitted the models with linear time and time as a categorical variable. The models were constructed with the first measurement week as the reference conditions so that positive coefficient estimates represent improved scores relative to baseline. For a one-arm pre-post design like this study, one must make the strong assumption that sites without an intervention would not change over time for the coefficients to be unbiased estimates of impact on implementation outcomes. Predicted means and 95% CI for each week are reported graphically. Distributions of residuals and plots of residuals vs. predicted values were examined to assess model assumptions. Non-parametric Spearman’s correlations with week and Kruskal–Wallis tests were used to assess the sensitivity of analysis conclusions to model assumptions. We also assessed the effects of missing dashboard data on time trend analyses using fully conditional multiple imputation (*N* = 25 imputations) (36). In addition, we conducted analyses to investigate how composite preparedness was associated with the number of activities and how these influenced the number of referrals via direct and indirect pathways (37, 38). Referrals, composite cessation activities, and composite preparedness measures are described above. The three variables were standardized to *z*-scores before analyses.

We used linear mixed model analyses with random site effects to assess relationships among preparedness, activities, and referrals. Four models were fitted (site random effect and residual error terms not shown):

Model A: Activities = *i* + *a* × Preparedness
Model B: Referrals = *i* + *b* × Activities
Model C: Referrals = *i* + *c* × Preparedness
Model D: Referrals = *i* + *b*′ × Activities + *c*′ × Preparedness

Estimated regression coefficients represent the change in the dependent variable in standard deviation units that are associated with a one standard deviation increase in the independent variable. Using coefficients from these models, we estimated unadjusted non-causal effects for Models A, B, and C. By including Model D, we were able to estimate the indirect effect of composite preparedness on composite referrals as mediated by composite activities as the product of the Model A preparedness coefficient, *a*, times the Model D activities coefficient, *b*′ (37, 38). To conduct the analysis, Week 1 preparedness was linked to Week 4 activities and referrals and by week for subsequent Weeks 12 and 36. Sensitivity to missing values was assessed by imputing missing preparedness and referrals with available nearest-neighbor values that were closest in time to the missing data for Site E (Week 12 for missing Week 4) and Sites F and G (Week 32 for missing Week 36). Statistical significance was set at *α* = 0.05, and analyses were conducted using SAS v9.4 (mixed, glimmix, causalmed, mi, and mianalyze). For this study of seven intervention-only sites, we did not adjust for multiple comparisons.

## Results

To test our hypotheses around the mechanism of the ATTOC intervention, we evaluated the impact on preparedness and activities in the intervention sites.

### Environmental Scan

**Figure 1:**
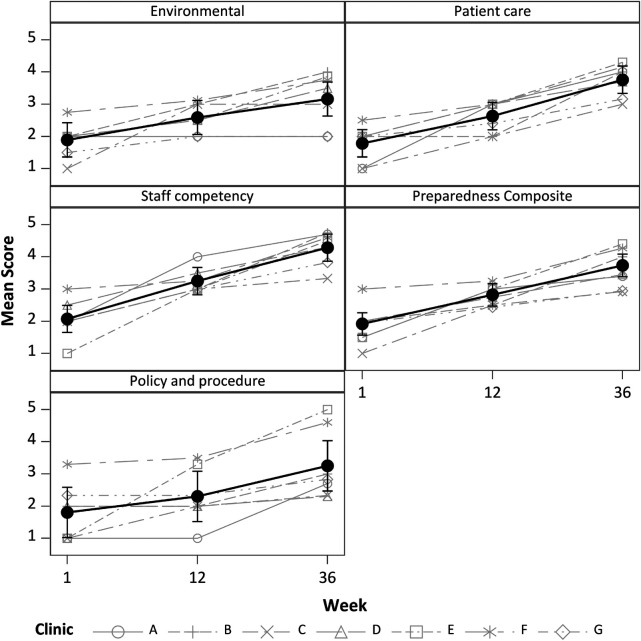
Scores for individual Environmental Scan components and for the composite score. Black symbols and whiskers are means and 95% CI. Gray lines are individual sites, which may overlap and be obscured. All Environmental Scan measures increased significantly after Week 1, *p* < 0.001 for all.

ES reports were collected for all sites and weeks. Scores were relatively low at Week 1 and increased significantly to Week 36 (Figure 1, *p* < 0.001 for all). At Week 36, three out of seven clinics had a composite preparedness score of at least 4.0. Staff competency had the highest score at Week 36 (4.3 ± 0.5), and the tobacco policy and procedures component had the most between-clinic variation in scores (SD = 1.1). Over the course of the 9-month intervention, improvement in scores had a linear trend (*p* < 0.001 for all) with no apparent leveling off after Week 12.

### ATTOC Dashboard

**Figure 2:**
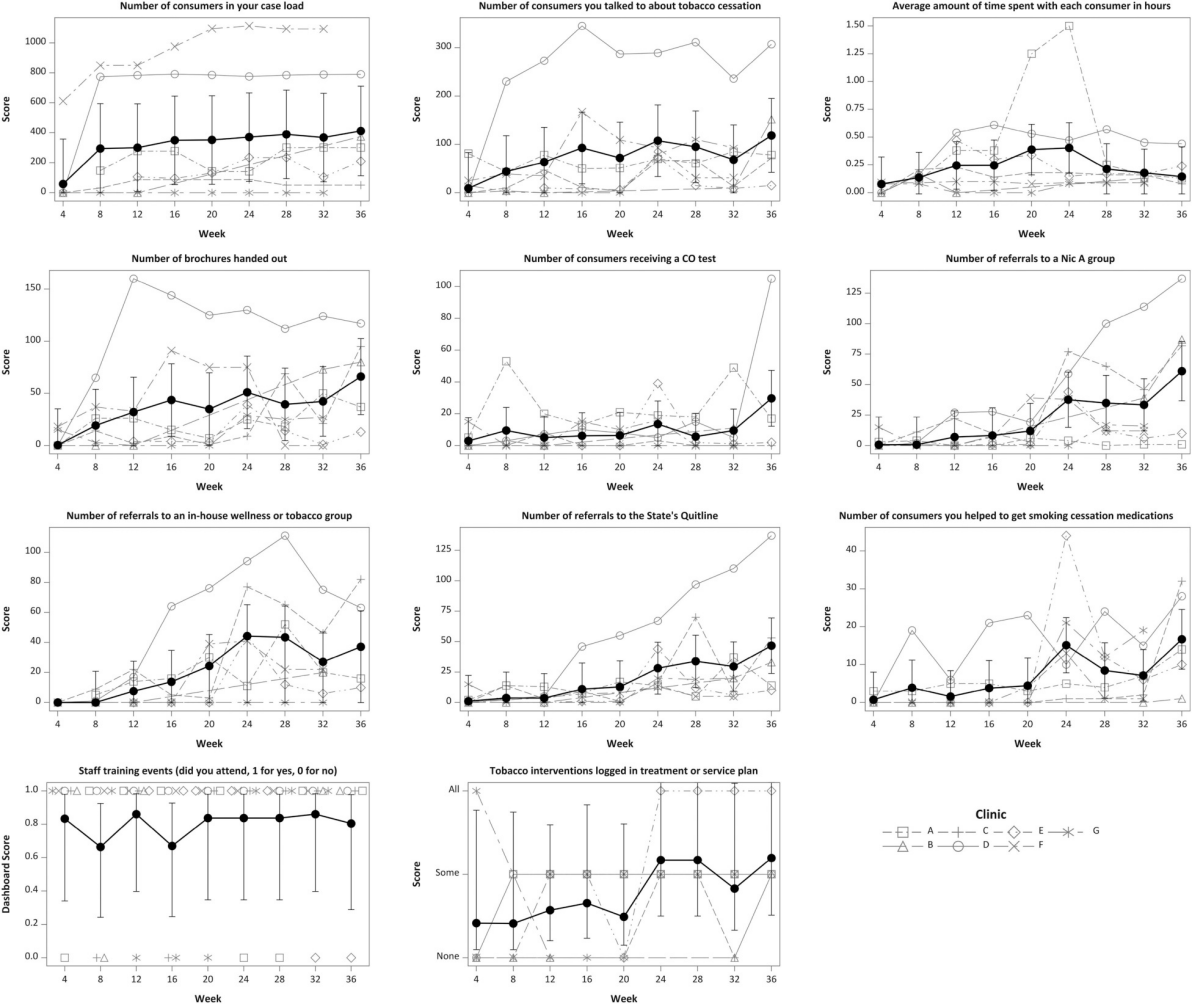
Dashboard items by week for seven participating intervention sites. Black symbols and whiskers are means and 95% CI. Gray lines are individual sites, which may overlap and be obscured or may be missing. Site B did not report data for Weeks 16–28, Site E is missing Weeks 4 and 8, and Sites F and G are missing Week 36.

Eight out of 11 ATTOC Dashboard items showed significant changes over time (Figure 2), including increased number of patients treated (*p* = 0.002), number of patients spoken to about tobacco cessation (*p* = 0.022), number of patients who received educational brochures (*p* = 0.034), and number of patients referred to a Nicotine Anonymous group (*p* < 0.001), an in-house wellness or tobacco group (*p* < 0.001), and state quitline (*p* = 0.012). In addition, there was an increase in tobacco use and TUD documentation in treatment plans (*p* = 0.008). The amount of missing data was variable for clinic-collected dashboard items. In contrast to the ES measures, nearly half of the dashboard items were missing for Clinic B (49%), Clinic E had 22% missing, Clinic G had 15% missing, and the remaining clinics had <5% missing data. The results from fully conditional multiple imputation confirmed the conclusions based on available case analyses. Examination of residuals revealed potential outliers and variances possibly increasing with predicted values. Spearman's correlation analyses between dashboard items and week remained significant for all except the number of patients treated (*p* = 0.053); however, Spearman's correlations did not account for repeated measures.

### Impact of preparedness and tobacco cessation activities on referrals

**Figure 3:**
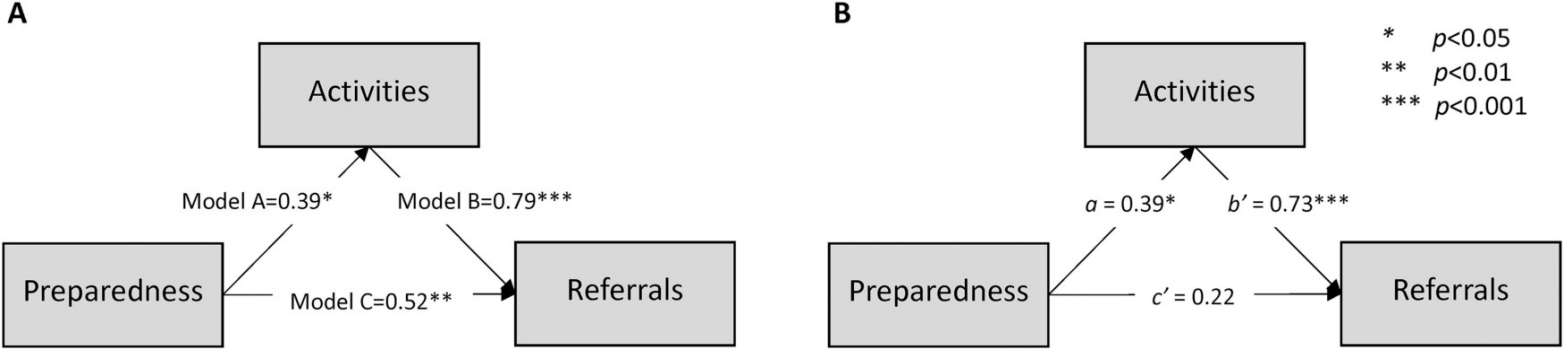
Proposed conceptual model for the impact of preparedness and tobacco cessation activities on referrals. **(A)** Unadjusted standardized coefficients from three separate generalized linear mixed model analyses: Model A, Model B, and Model C. **(B)** Coefficients from Model A (*a*) and Model D (*b*′ and *c*′). The indirect effect of preparedness mediated by activities is *a* × *b*′ = 0.29 (SE = 0.12, *p* = 0.021).

We hypothesized that increasing preparedness and cessation activities would be predictive of referrals (Figure 3A). In the separate mixed model analyses, we noted significant unadjusted associations with the site-preparedness and the number of activities undertaken by the sites, including providing brochures and discussing tobacco cessation services. Both composite preparedness (Model C = 0.53 *p* = 0.006) and composite activities (Model B = 0.79; *p* < 0.001) were significantly associated with the number of composite referrals. We also found that the average number of activities increased significantly more when average preparedness was higher (Model A = 0.39; *p* = 0.018). Coefficients from our multivariable Model D are shown in Figure 3B. The adjusted association between referrals and preparedness was *c*′ = 0.20 (95% CI: −0.09 to 0.49, *p* = 0.158), and the activities coefficient was *b*′ = 0.73 (95% CI: 0.44–1.01, *p* < 0.001). Model D explained 64% of the variation in the number of referrals. The coefficient in Figure 3B is the same as in Model A in Figure 3A. The indirect effect pathway from preparedness through composite activities to referrals was *a* × b′ = 0.29 (SE = 0.12, *p* = 0.021), which accounted for 54% of the total effect of preparedness on the number of referrals (Model C = 0.52; 95% CI: 0.18–0.87, *p* = 0.005). The results from sensitivity analyses were not substantially different from the available data analyses.

## Discussion

Tobacco use among individuals with SMI is common. Changing the organizational culture to integrate evidence-based treatments for TUD will be vital to address this health disparity. This paper confirms that support for preparedness and activities leads to increased number of patient interventions on-site (assessments, discussions with patients about tobacco use, brochures created and given, and CO monitoring) and referrals within the site and to community interventions (referrals to a Nic A group, an in-house wellness or tobacco group, and state quitline and number of patients obtaining TUD smoking cessation medications) during the 10 session, 9-month ATTOC model intervention.

This study also demonstrated the process by which organizational change (i.e., increased preparedness, increased activities, and increased number of referrals) can lead to implementation of evidence-based interventions for TUD in behavioral health settings. The same organizational ATTOC approach was used at each site; however, each site determined its change goals in three critical areas: (1) patient care and treatment, (2) staff training and staff smoking, and (3) their campus becoming tobacco-free in total or in a restricted manner. The proposed work builds on the substantial work supporting the feasibility and impact of ATTOC and is part of a larger study attempting to validate the ATTOC model for organizational change in a randomized control trial, building on previous work for a single-arm evaluation (18, 34). Informed by implementation science and organizational change theory, this paper focused on elucidating the mechanistic pathways to changing organizational culture and can be generalized to culture changes needed to enhance clinical outcomes through the integration of evidence-based treatments for the intervention sites.

This study found significant improvements through the ATTOC Environmental Scan assessment in critical areas of the clinic, including staff training, restrictions on tobacco use or fully tobacco-free campuses, new policies, integration of new evidence-based treatments (medications and psychosocial and community-based approaches), communication tools, and patients engaged in assessment and treatment. As described in the results, there was a significant change across the sites for the Environmental Scan composite preparedness score and for each of the four core individual areas (environmental, patient care, staff competency and recovery, and policy/procedures) of the ES.

The findings reported from the 11 ATTOC Dashboard items showed significant improvements in the number of patients treated, talked with about tobacco cessation, provided educational brochures, and referred to Nic A and in-house wellness or tobacco groups and state quitline, as well as documentation in treatment plans. Dashboard items were not available for all weeks from some sites (Figure 2), which increased variability when evaluating trends. Changes in preparedness and cessation activities were predictive of the number of referrals (Figure 3), with 54% of the preparedness impact due to its positive association with increasing activities. Future reports will focus on the clinical outcomes of the patients, whereas this report focuses on the ATTOC intervention being able to support the needed organizational changes.

Having frontline staff with skills to discuss TUD and provide evidence-based treatments for patients with varying levels of quit motivation appears critical to enhancing the integration of evidence-based treatments and improving TUD treatment engagement and outcomes. As such, staff were prepared to confidently provide general information and connect patients to resources through phone, online, internal tobacco cessation specialists, or external community resources. The ongoing nature of the training appears to support the culture change and the impact of more patients receiving the interventions. A potential barrier to addressing TUD with patients and building efficacy, confidence, and competence is the staff member's own challenges with quitting smoking. Addressing this within the ATTOC intervention appears to help increase the number of patients receiving care, as noted in the larger study (18). Stigma can impact staff members (and patients) who may be dealing with a sense of shame, embarrassment, or guilt, resulting in a lower likelihood of helping their patients address tobacco use. ATTOC addressed this concern directly in the training and also by supporting tobacco cessation among staff members by encouraging the organization to either cover costs or reduce co-pays for treatment and providing them time off for such treatment. Changes by the organization's CEO, clinical leaders, and frontline clinicians were noticed and assessed in the Environmental Scan, and these changes appear to be associated with providing more TUD patient care. The changes in agency readiness were also reflected by staff having been able to reflect on other times they were successful on large organizational changes, their values of the importance of the recovery and wellness in the organization, and their personal mission, and through the support of the local champion. A strong champion demonstrates leadership, management, and effective communication, knows the organizational change process, and has the ability to collaborate and support all aspects of the ATTOC processes, phases, and steps.

The study has potential implications for impacting clinical practice, healthcare policies, and future research directions. First, the findings demonstrate how the ATTOC intervention helped agencies follow both the PHS guidelines for clinical practice changes and organizational changes (i.e., clinical system policies, screenings and assessments, and trainings). By doing so, the ATTOC model led to sites making the organizational changes recommended and by having more clinical services and better trained staff than the control study [see the main study findings of Schnoll et al. (18)], which builds capacity in the system through organizational change. These expanded clinical services were advanced because of the staff trainings, mentoring of champions who were members of the agency and led the organizational change effort with the support of the ATTOC consultants, the development of new patient educational materials, provision of CO meters for clinical programs, trained staff on screenings and assessment, starting a quitting group or group for lower motivated patients to increase their motivation (i.e., learning about health living), and medication management training. From a policy perspective, this study builds on the PHS guidelines and has implications for changes in the Joint Commission standards for behavioral health care organizations to ensure safe, high-quality treatment. The Joint Commission standards are stronger for general medical facilities, and this study suggests that if there were higher standards in behavioral health settings that were similar to general medical settings, it would help facilitate needed organizational change as well as enhanced clinical services. Finally, future research should assess organizational change in both the intervention and control groups and further increase the sample size. Additional patient care outcomes might also be added in both the intervention group and the control group, including assessing CO levels. Future research might incorporate a focus on implementation costs and cost–benefit that can further strengthen the evidence for implementing the ATTOC model and also align with prevention research, which demonstrates the return on investment for these initially higher costs. Furthermore, using the Project Extension of Community Health Outcomes approach (39) could be a method to increase the sample size of sites dramatically and be a potential opportunity to deliver the training with low cost and with maximum reach, allowing for a group effect of the “all teach all learn” principle, rather that one program being supported in isolation.

We acknowledge that organizational change requires time; however, in the current study, by focusing on specific implementation indicators such as preparedness, activities, and referral, we were able to show positive organizational change. As one might expect, changing tobacco policy and procedures takes longer in some organizations due to the amount of discussion required and formal processes for getting policies added or changed. For example, one site needed 12 weeks to get policies modified. Addressing tobacco is a culture change, and in one site, this was more difficult, and there was limited change in staff competency, smoking, and attitudes that addressing tobacco was important. The policies and procedures at the site are influenced by both staff attitudes about addressing tobacco as well as staff recovery rates. In previous ATTOC studies, these policies and procedures can be more difficult at sites that have a high rate of smokers, limited resources for staff to quit, or when there are other competing organizational demands.

The study results should be interpreted in the light of limitations. First, since the control sites did not receive implementation support, the organizational change data (Environmental Scan and ATTOC Dashboard) were not collected in the control sites. For a one-arm pre-post design like this study, there is limited generalizability, and one must assume that sites without an intervention would not change over time for the coefficients to be unbiased estimates of impact on implementation outcomes. Second, there were some differences in client and staff characteristics between ATTOC sites and our control sites (18), suggesting caution in generalizing these results to all community mental health sites. Finally, we acknowledge the missing data and performed additional model diagnostics to ensure that there was no evidence of non-normality or heteroscedasticity due to the missing data. Future implementation efforts can focus on reducing missing data when collecting implementation outcomes and be supported through qualitative methods of inquiry.

## Conclusion

The sites that participated in ATTOC showed improvement in the provision of evidence-based treatments for tobacco use in outpatient mental health treatment settings and enhanced campus tobacco use restrictions, staff training, staff TUD recovery, and TUD clinical documentation, policies, and new signage/educational materials for patients. It is important to include patients, staff, and environmental assessment to promote cultural change in tobacco cessation programs. Studying the indicators and mechanisms of implementation in the intervention sites provided much-needed information on the organizational change process and its impact.

A collaborative effort must be made to fight stigma that impacts vulnerable populations, such as individuals with SMI. Addressing environmental factors, including challenging long-held myths and beliefs about tobacco use, becomes critical in tobacco cessation treatment programming, procedures, and policies. And while the efficacy of tobacco cessation interventions has improved the lives of many populations across the globe, broader, innovative approaches that target underserved or vulnerable populations are needed to address various socioecological factors that contribute to tobacco use. Furthermore, interventions must move beyond only targeting TUD and move toward the promotion of wellness and recovery paradigms that address comprehensive healthy ways of living, including a system of social support, among individuals with SMI. Treatments that target mental health systems may show greater promise for tobacco cessation than individual-level interventions alone.

## Data Availability

The raw data supporting the conclusions of this article will be made available by the authors, without undue reservation.
